# Age-Related Patterns in Human Myeloid Dendritic Cell Populations in People Exposed to *Schistosoma haematobium* Infection

**DOI:** 10.1371/journal.pntd.0001824

**Published:** 2012-09-27

**Authors:** Norman Nausch, Delphine Louis, Olivier Lantz, Isabelle Peguillet, François Trottein, Isobel Y. D. Chen, Laura J. Appleby, Claire D. Bourke, Nicholas Midzi, Takafira Mduluza, Francisca Mutapi

**Affiliations:** 1 Institute of Immunology and Infection Research, Centre for Immunity, Infection and Evolution, School of Biological Sciences, Ashworth Laboratories, University of Edinburgh, Edinburgh, United Kingdom; 2 Institut Curie, Département de Biologie des Tumeurs, Paris, France; 3 Centre d'Investigation Clinique IGR-Curie, CIC-BT-507, Paris, France; 4 Unité Inserm 932, Institut Curie, Paris, France; 5 Center for Infection and Immunity of Lille, Inserm U 1019, CNRS UMR 8204, Université Lille Nord de France, Institut Pasteur de Lille, Lille, France; 6 National Institute of Health Research, Causeway, Harare, Zimbabwe; 7 Department of Biochemistry, University of Zimbabwe, Mount Pleasant, Harare, Zimbabwe; René Rachou Research Center, Brazil

## Abstract

**Background:**

Urogenital schistosomiasis is caused by the helminth parasite *Schistosoma haematobium*. In high transmission areas, children acquire schistosome infection early in life with infection levels peaking in early childhood and subsequently declining in late childhood. This age-related infection profile is thought to result from the gradual development of protective acquired immunity. Age-related differences in schistosome-specific humoral and cellular responses have been reported from several field studies. However there has not yet been a systematic study of the age-related changes in human dendritic cells, the drivers of T cell polarisation.

**Methods:**

Peripheral blood mononuclear cells were obtained from a cohort of 61 Zimbabwean aged 5–45 years with a *S. haematobium* prevalence of 47.5%. Two subsets of dendritic cells, myeloid and plasmacytoid dentritic cells (mDCs and pDCs), were analyzed by flow cytometry.

**Findings:**

In this population, schistosome infection levels peaked in the youngest age group (5–9 years), and declined in late childhood and adulthood (10+ years). The proportions of both mDCs and pDCs varied with age. However, for mDCs the age profile depended on host infection status. In the youngest age group infected people had enhanced proportions of mDCs as well as lower levels of HLA-DR on mDCs than un-infected people. In the older age groups (10–13 and 14–45 years) infected people had lower proportions of mDCs compared to un-infected individuals, but no infection status-related differences were observed in their levels of HLA-DR. Moreover mDC proportions correlated with levels of schistosome-specific IgG, which can be associated with protective immunity. In contrast proportions of pDCs varied with host age, but not with infection status.

**Conclusions:**

Our results show that dendritic cell proportions and activation in a human population living in schistosome-endemic areas vary with host age reflecting differences in cumulative history of exposure to schistosome infection.

## Author Summary

A characteristic feature of most helminth infections is the convex age infection profile, where infection levels rise to peak in early childhood and decline in adulthood, a pattern thought to result from the development of protective acquired immunity. Thus, several investigations characterizing protective responses to inform vaccine research have focused on responses present in older people, who despite continued exposure to infection carry little or no infection. To date, such studies have identified key responses which are correlates of resistance. However, there is a paucity of information on cell types that are mediators rather than effectors of the immune responses. One such group where there are limited studies in human schistosome infections is dendritic cells which are important for the polarizations of CD4+ T cell responses. Therefore, we characterized the age profile of dendritic cells in Zimbabweans exposed to *Schistosoma haematobium* infection. We found an age-related pattern in the proportions of myeloid dendritic cells (a subset of dendritic cells) in this population. Furthermore, in the case myeloid dendritic cells, the age profile differed between schistosome infected and un-infected people. Thus our study suggests that activation and migration of myeloid dendritic cells also develop in an age-related pattern consistent with the cumulative history of exposure to schistosome parasites.

## Introduction

*Schistosoma haematobium* helminth parasites cause urogenital schistosomiasis which affects about 112 million people mainly in rural areas of subtropical countries [1]. Infections with *S. haematobium* are most common in school-age children. Populations in endemic areas show a characteristic age-infection profile with infection levels increasing to peak in early to late childhood, typically around 9–15 years and then declining in adulthood [2]–[4]. This profile is believed to be largely reflective of the gradual development of protective acquired immunity reducing re-infection levels [2], [5], [6]. The consequence of this age profile is that individuals with comparable infection levels who have resided in a schistosome endemic area since birth (e.g. egg negative children *versus* egg negative adults) can differ significantly in their immune response against the parasite and thus their levels or resistance to re-infection. Several studies characterizing human immune responses to schistosome infections have shown age related differences in antibody levels [7]–[9], plasma cytokines [10], parasite-specific cytokines [11] and regulatory T cell proportions [12]. This concept is supported by theoretical modelling of the development of acquired immunity, which predicts that correlations between immunological responses and infection is positive in younger age groups and subsequently decreasing and potentially turning in a negative correlation [13]. Moreover this pattern is not restricted to schistosome infections, but is a characteristic of immune responses associated with protection from a variety of helminth species in endemically-exposed populations [14], [15].

As investigations of the nature and development of protective acquired immunity progress, there has been a move to decipher the mechanisms and pathways behind these observed age-related patterns. Human field studies show that the immune response against schistosomes is characterized by a very complex interaction of T_H_1, T_H_2 and regulatory responses [10]–[12], [16]–[18] with differences between natural human infections and experimental mouse models [19], [20]. Nevertheless, the induction of a T_H_2 response is important in the immune response against schistosomes [21] and in particular for the development of protection [22]. As a major antigen-presenting cell population dendritic cells (DCs) are responsible for acquiring, processing and presenting parasite antigens to T cells and the latter interaction leads to activation and polarisation of the acquired immune response. The importance of DCs in the induction of the T_H_2 immune responses in the context of schistosome infections has been recently highlighted in an experimental mouse model [23]. This study indicates that DCs are required for the induction and development of T_H_2 responses during schistosome infections supporting other studies showing the importance of DCs in the context of T_H_2 induction [24]–[27].

It has been shown that schistosome derived compounds can activate dendritic cells by toll-like receptors (TLR)-2 and 3 [28], [29] and early T_H_2-promoing activities can be diminished by TLR-3 [30]. Overall studies are rare addressing the role of the innate immune cells in the immune response against schistosomes [31].

In particular, studies analysing DCs directly from humans exposed to schistosomes are limited. The first study addressing this question, published by Everts and colleagues in 2010, elegantly showed changes in DCs during chronic schistosomiasis (*S. haematobium*) [32] suggesting that chronic schistosomiasis can suppress DCs, which might play a role in immune modulation by schistosomes.

Peripheral blood DCs can be divided in two major subsets [33], [34], myeloid dendritic cells (mDC; or conventional DC) and plasmacytoid (pDC). Myeloid DCs express CD11c, but low levels of CD123 and have a more pronounced role in antigen processing and initiation of T cell response [35]. Plasmacytoid DCs express CD303 (BDCA-2), ILT7, high levels of CD123 (IL-3Rα), but are negative for the classical marker CD11c [33], [36]–[38]. These pDCs play a critical role in anti-viral immunity as well as in immune tolerance [39], [40]. Everts *et al*'s [32] study focusing on people aged 17–39 years showed that the frequencies of mDCs and pDCs are reduced in infected people compared to un-infected and that mDCs are functionally impaired in response to toll-like receptor ligands and in driving T cell responses *ex vivo*. Since in most schistosome endemic areas people are exposed to infection from as young as 6 months old [41], and may already be carrying heavy infections within the first decade of life [8], these early infection events may have a profound effect on the proportions of the DCs present in the host. Characterising the role of these cells during natural immune responses to helminth infection in the different age groups is vital for vaccine development, since people targeted by anti-schistosome vaccines in endemic areas will have been previously exposed to infection and their cellular immune responses will already be primed by repeated exposure to parasite antigens. In this context variations in antigen presenting cell populations, including DCs, might directly affect the efficacy of vaccination in different age groups. Lessons from the discontinued human hookworm vaccine trials, illustrate the importance of characterising existing natural immune responses in endemic populations and designing vaccine to avoid undesirable pathological outcomes of vaccination [42]. Therefore, the aim of this study was to characterise the relationship between age and DCs during natural schistosome infections and determine if these patterns are affected by host infection status across different age groups reflecting different dynamics in the acquisition and loss of schistosome infection.

## Methods

### Study population and parasitology

This study was performed in Chipinda village which is located in the Mashonaland East Province in Zimbabwe (31°94′E; 17°67′S). The village was selected because health surveys regularly conducted in the region showed little or no infection with soil-transmitted helminths (STH) and a low *S. mansoni* prevalence (<2%), which elicit immune responses that cross-react with those against *S. haematobium* [43], [44], [45]. The low STH and *S. mansoni* prevalence is consistent with earlier surveys in this area of Zimbabwe [46]–[48]. Villagers are subsistence farmers who have frequent contact with water sources posing a risk of infection (as assessed by questionnaires) due to insufficient safe water provision and low coverage of sanitation facilities as is typical in rural Zimbabwe. Drinking water is collected from open wells while bathing and washing is conducted in perennial rivers surrounding the village. This area has not been included in any Schistosome Control Programmes and therefore participants had not received any anti-helminthic treatment for schistosomiasis or other helminth infections prior to this study. Thus their natural immune responses could be assessed in the absence of drug-altered schistosome-specific responses [49], [50]. Urine and stool samples were collected on three consecutive days and examined microscopically for *S. haematobium* (urine filtration, Mott method [51]) or *S. mansoni* and intestinal helminths (Kato-Katz method [52]) respectively using standard procedures. Participants were screened for malaria by microscopic examination of Giemsa stained blood smears and HIV status was determined by immunochromatography (DoubleCheckGold™ HIV 1&2, Orgenics) with HIV positive samples subsequently re-tested by a second rapid assay (Determine HIV 1/2 Ag/Ab Combo, InvernessMedical) to confirm HIV status [53]. Study participants had to meet the following criteria: (1) be life-long residents in this area (assessed by questionnaires) so that age would be a proxy for duration of exposure to *S. haematobium* infection, (2) should not have received anti-helminthic treatment prior to this study, (3) should have provided at least two urine and two stool samples on consecutive days for parasitological diagnosis, (4) should have tested negative for intestinal helminths including *S. mansoni* to focus on single infections with *S. haematobium*, (5) should be negative for HIV and malaria (Prevalence of both HIV and malaria were too small for inclusion in the statistical analysis (HIV prevalence in Chipinda village was 8% and no-one was positive for malaria infection at the time of sampling) and (6) have provided a sufficient volume of blood to isolate peripheral blood mononuclear cells (PBMC). The selected cohort comprised 61 individuals and details to the cohort are provided in Table 1.

**Table 1:** Study population.

				*S. haematobium*	
Age group	N	mean age in years (range)	Male/female	Prevalence in % (95% CI)	Mean egg count ± SEM (range)
**Total**	61	14.1 (5–45)	27/34	47.5 (35.6–59.9)	51.6±19.3 (0–859)
**Group 1**	17	7.6 (5–9)	8/9	35.3 (15.9–54.8)	78.1±51.4 (0–859)
**Group 2**	23	11.1 (10–13)	8/15	52.2 (36.0–68.5)	47.7±25.9 (0–571)
**Group 3**	21	22.6 (14–45)	11/10	52.4 (35.0–69.9)	34.5±26.0 (0–550)

SEM – Standard error of mean, CI – Confidence interval.

### Ethical statement

Permission to conduct the study in the region was obtained from the Provincial Medical Director and institutional and ethical approval was received from the University of Zimbabwe's Institute Review Board and the Medical Research Council of Zimbabwe respectively. Only compliant participants were recruited and they were free to drop out at any point during the study. At the beginning of the study, participants and their parents/guardians (in case of children) had the aims and procedures of the project explained fully in the local language, Shona, and written consent was obtained from participants and parents/guardian before parasitology and blood samples were obtained. After collection of all samples, all participants were offered anti-helminthic treatment with the recommended dose of praziquantel (40 mg/kg of body weight).

### Blood collection and isolation of PBMC

Depending on age of the participants up to 25 ml of venous blood was collected in heparinized tubes of which approximately 5 ml was used for serological assays as well as microscopic detection of malaria parasites. The remaining blood was used for the isolation of peripheral blood mononuclear cells (PBMC) through density gradient centrifugation using Lymphoprep (Axis-Shield, Cambridgeshire, UK). These PBMC were subsequently enumerated, cryo-preserved in 10% DMSO, 90% fetal calf serum and stored in liquid nitrogen in Zimbabwe prior to shipping to Edinburgh in dry shippers for assaying.

### DC phenotyping

Thawing of cryo-preserved PBMC was performed by rotating cryovials in a 37°C water bath until a small crystal was remaining in the cell suspension. Cells were then slowly re-suspended in RPMI 1640 supplemented with 10% FCS, 2 mM L-glutamine and 100 U Penicillin/Streptomycin (all Lonza, Verviers, Belgium). Cells were washed twice with media, counted and viability assessed using trypan blue (Sigma-Aldrich, Dorsert, UK). The median viability of the PBMCs was 71.4% which is within the range of published values [54]. In addition it was confirmed that including a viability marker did not change analysis of subsets. Afterwards, cells were washed with Dulbecco's-PBS (Lonza) and surface stained with the following antibodies: Qdot-605-conjugated anti-CD14 (clone TUK4, Invitrogen), FITC-conjugated anti-CD11c (clone Bu15, Invitrogen), APC-H7-conjugated anti-HLA-DR (clone L243, BD Biosciences), PE-Cy5. anti-CD123 (clone 9F5, BD Biosciences) and V450 BD Horizon-conjugated anti-CD86 (clone 2331, BD Biosciences), PE-conjugated anti-BDCA-2 (clone AC144, Miltenyi Biotec) and APC-conjugated anti-BDCA-4 (clone AD5-17F6, Miltenyi Biotec). Stained cells were acquired on a FACSCantoII (BD Biosciences) and analyzed using FlowJo software software (TreeStar, USA).

### Antibody ELISAs

Serum antibody levels were measured by enzyme-linked immunosorbent assays (ELISA) following established protocols [8], [55]. Whole worm homogenate (WWH) was obtained from the Theodor Bilharz Research Institute (Giza, Egypt). In short, microtiter plates were coated overnight at 4°C at 10 µg/ml. Serum samples were diluted at 1∶20 for WWH-IgE and 1∶100 for WWH-IgG and IgM and incubated for 2 hours at 37°C. Horse-radish peroxide conjugated antibodies were diluted 1∶1000 for IgG, IgE and IgM and incubated for 1 hour at 37°C. ELISAs were developed using ABTS (Southern Biotech) and stopped after 15 minutes for IgG and IgM, and 30 minutes for IgE. Absorbance was read at 405 nm. Serum was available from 45 out of 61 individuals.

### Statistical analysis

The proportions of DC cell subsets were square root arcsine transformed whereas expression levels of HLA-DR and CD86 were square root transformed to allow the use of parametric tests in subsequent analyses [56]. To analyse which factors influence the proportion of DC subsets, a univariate analysis of variance using sex (male/female), host age groups (group 1: 5–9 years; group 2: 10–13 years; group 3: 14+ years) and infection status (un-infected = 0 mean egg count per 10 ml and infected >0 mean egg count per 10 ml; all variables categorical) as independent variables was performed. The three different age groups were selected to reflect epidemiological groupings (i.e. where infections are acquired, peak and decline) by age and infection intensity and to obtain comparable sample sizes between the groups. Post hoc tests between infected and un-infected individuals were conducted in each age group. In addition post hoc analysis was performed to determine differences in pDCs between the three age groups. To determine if the mDC populations showed an age profile consistent with those for protective immune responses as predicted by quantitative studies [13], correlation analyses between infection intensity (log_10_ (mean egg count+1) transformed) and proportions of mDCs was conducted after allowing for the effect of sex. The correlation coefficients were then tested for homogeneity using the Fisher's r-to-z transformation [57]. Correlation analyses between WWH-specific IgG and DC cell subsets were performed after allowing for the effects of sex, age and infection intensity. All statistical tests were conducted using the software package SPSS v14 and p values were taken to be significant at p<0.05.

## Results

### Epidemiology of study population

**Figure 1:**
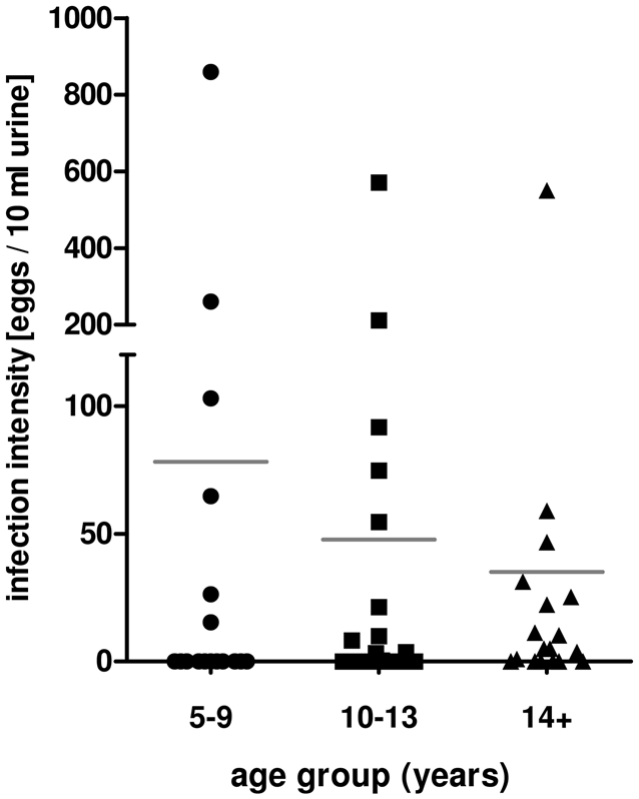
Age – infection profile of the study population. The study population was divided into three age groups: 5–9 years (N = 17), 10–13 years (N = 23) and 14+ years (N = 21). Mean infection intensities from at least two urine samples for each individual are given. The mean for each group is indicated.

**Figure 2:**
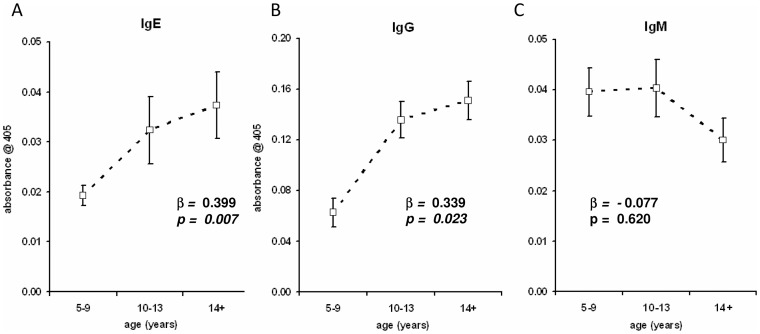
Schistosome-specific antibody profiles. Serum levels of WWH-specific IgE (A), IgG (B) and IgM (C) were analysed by ELISA. Obtained data are presented after diving into the three indicated age groups. Correlation coefficient b and significant values are indicated. The effect of sex has been statistically allowed for.

This study was designed to focus on the immune modulation in a population exposed to *S. haematobium*. The northeast part of Zimbabwe is endemic for *S. haematobium* with many regions having a moderate to high prevalence of *S. haematobium*, but low prevalence of *S. mansoni* and soil-transmitted helminths [47], [48]. The overall prevalence in Chipinda was (39%). This is defined by the World Health Organisation (WHO) as a moderate transmission area [58]. The overall prevalence of *S. haematobium* in the selected study population (N = 61) was 47.5% which is higher but not significantly different from (χ^2^ = 1.688, df = 1, p = 0.097) the village prevalence of 39% and still within the WHO definition of moderate transmission. The difference in infection prevalence between the youngest age group (35.3%) compared to more than 50% in the older age groups was not significant (χ^2^ = 0.806, df = 1, p = 0.185; details in Table 1). However, youngest individuals (5–9 years) who were infected carried high infection levels (Figure 1 and Table 1). Individuals aged 10–13 years still showed high infection levels, but with a higher prevalence than the first age group. In contrast, in the oldest age group (14–45 years), the prevalence remained high, but most individuals show lower infection intensities (Figure 1). The consequence of the infection profile is that individuals who are life long residents in the area and have never received treatment with anti-helminthic drugs (see selection criteria in Methods) differ in their cumulative histories of exposure despite carrying comparable infection intensities. This was supported by an analysis of the serum levels of adult worm specific (whole worm homogenate – WWH) antibody levels. As shown in Figure 2A and B levels of WWH-specific IgE and IgG, which are associated with history of infection and resistance to infection [59]–[61], increase significantly with age. In contrast IgM against WWH as marker of current infection starts to decrease in the oldest age group (Figure 2C).

### Characterization of mDCs and pDCs in total study population

**Figure 3:**
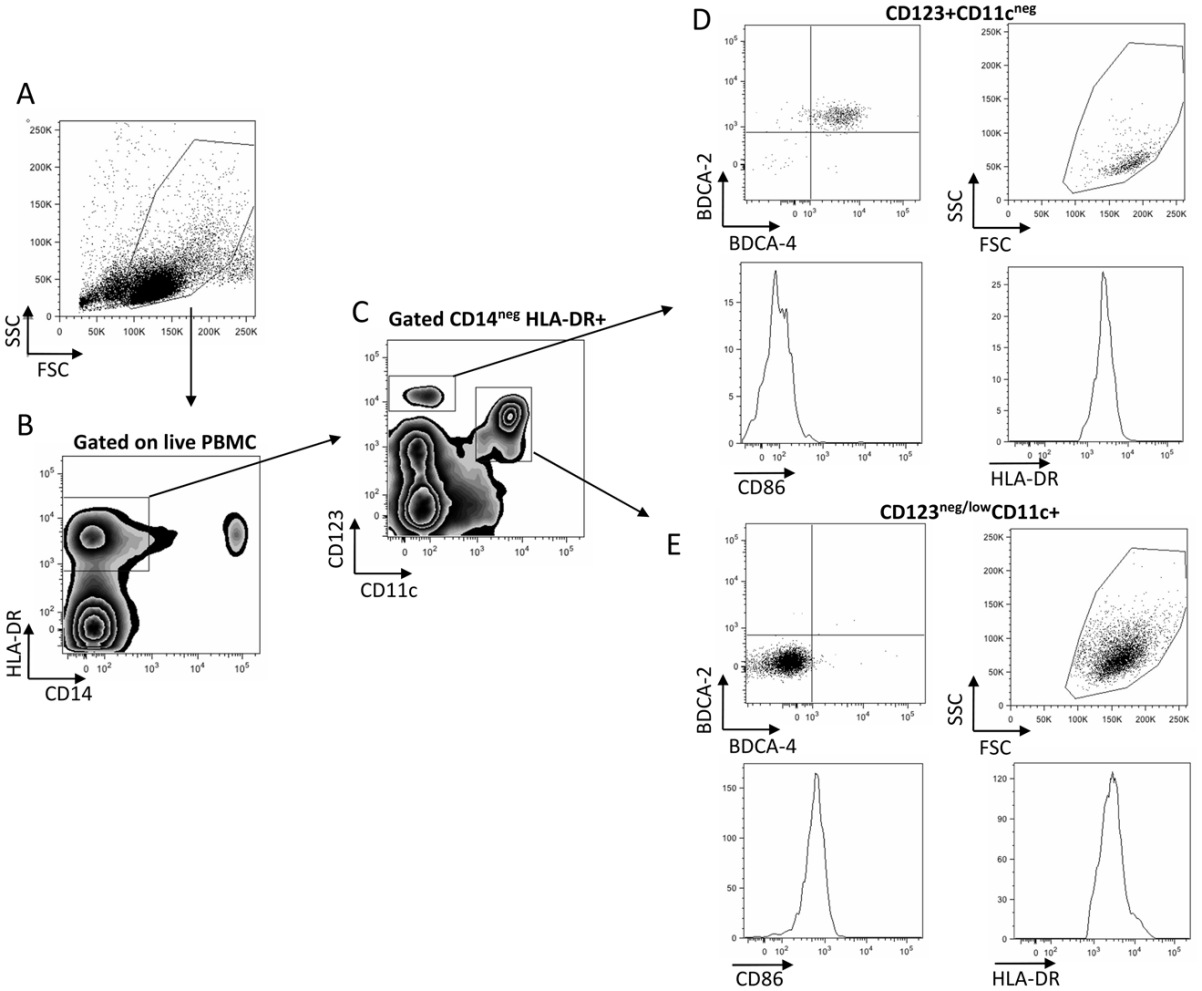
Phenotyping DCs. Live PBMCs (A) are gated on CD14^neg^HLA-DR+ cells (B) which subsequently are analysed for the expression of CD11c and CD123 (C). CD123+CD11c^neg^ (D) and CD123^neg/low^CD11c+ (E) were subsequently analysed for the expression levels of BDCA-2/CD303 and BDCA-4/CD304, CD86 and HLA-DR. Presented plots are an example and derived from a 5 year old female individual.

To analyze mDCs and pDCs, PBMC were gated on CD14^neg^HLA-DR+ cells (Figure 3A, B). Gated cells were stained with CD123 and CD11c to distinguish mDCs (CD11c+CD123^neg/low^) from pDCs (CD123^hi^CD11c^neg^; Figure 3C). To further verify specificity of pDC gated CD123^hi^CD11c^neg^ were analysed for the expression of BDAC-2/CD303 and BDCA-4/CD304 [62] and granularity of gated cells was analysed in an FSC/SSC plot (Figure 3D). Therefore other cell types such as basophiles or B cell potentially able to express CD123 are excluded from the analysis. mDC did not express either BDCA-2 or BDCA-4 (Figure 3E). Subsets were expressed as percentages of PBMC or as percentage of CD14^neg^HLA-DR+ cells. Both subsets were subsequently analysed for the expression of CD86 (Figure 3D, E) and levels of HLA-DR within the two different DC populations as indicators of DC activation status and their ability to present antigen and co-stimulatory signals to T cells.

**Figure 4:**
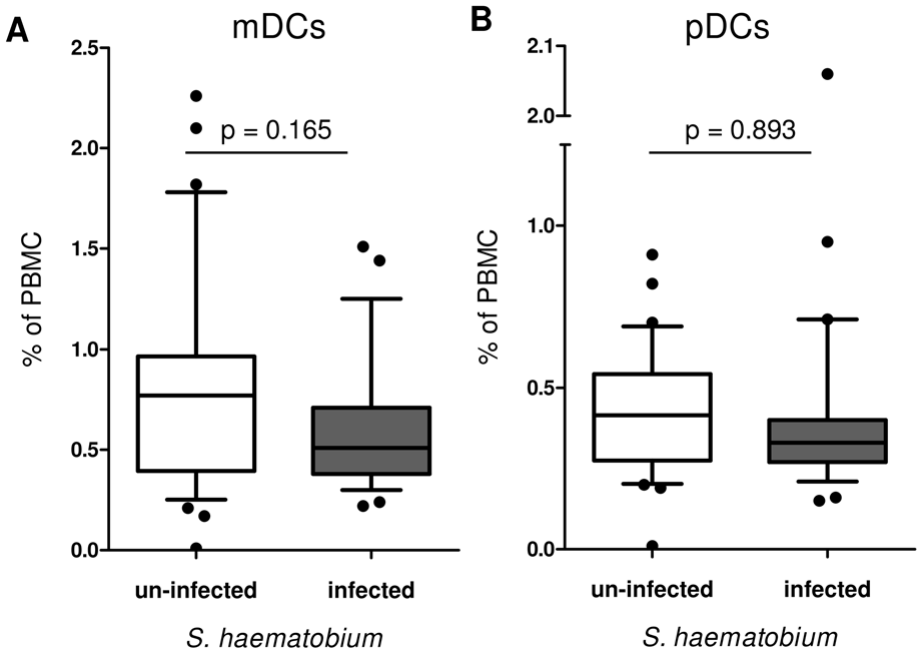
Proportions of DCs in the study population. PBMC (N = 61) were analysed for proportions of (A) CD123^neg/low^CD11c+ mDCs or (B) CD123^hi^CD11c^neg^ pDCs (expressed as % of PBMC). Data of the total population were divided in un-infected (N = 32, white box) and infected individuals (N = 29, gray box). Data are presented as Whiskers boxplot (10–90 percentiles). Differences were analysed by univariate analysis after accounting for the effect of sex.

When the study population was portioned by infection status (un-infected *versus* infected), neither mDCs (Figure 4A) nor pDCs (Figure 4B) showed a significant difference between both populations. Comparable results were obtained if DC subsets were expressed as percentage of CD14^neg^HLA-DR+ (data not shown). Myeloid DCs showed a significant association with sex (Table 2), in which female had slightly higher proportions than males. This was then statistically accounted for in all subsequent analyses.

**Table 2:** Effects of sex, age group and infection status on mDC and pDC proportions and levels of HLA-DR, CD86 expression on both subsets.

Dependent variable		Proportions of DCs			HLA-DR expression			CD86 expression		
DC population	Explanatory variable	df	F – value	p – value	df	F – value	p – value	df	F – value	p – value
***mDC***	sex	1, 60	4.659	**0.35**	1, 60	2.049	0.158	1, 60	0.297	0.588
	age group	2, 60	0.646	0.528	2, 60	0.068	0.934	2, 60	0.064	0.938
	infection status	1, 60	3.392	0.071	1, 60	0.372	0.544	1, 60	0.424	0.518
	Age group * infection status	2, 60	11.963	**<0.001**	2, 60	3.865	**0.027**	2, 60	0.763	0.471
***pDC***	sex	1, 60	2.509	0.119	1, 60	2.240	0.140	1, 60	0.015	0.902
	age group	2, 60	3.544	**0.036**	2, 60	0.075	0.928	2, 60	0.092	0.912
	infection status	1, 60	1.237	0.271	1, 60	0.842	0.363	1, 60	1.184	0.281
	Age group * infection status	2, 60	0.074	0.929	2, 60	1.246	0.296	2, 60	1.267	0.290

Results from univariate analyses of variance determining the effect of host sex, age group and infection status. Variables with significant influence are highlighted in bold. df –degree of freedom. For mDCs and pDCs proportions of PBMC were analysed, whereas the MFI was used for HLA-DR and CD86 expression.

### Age dependent pattern of mDCs and pDCs

**Figure 5:**
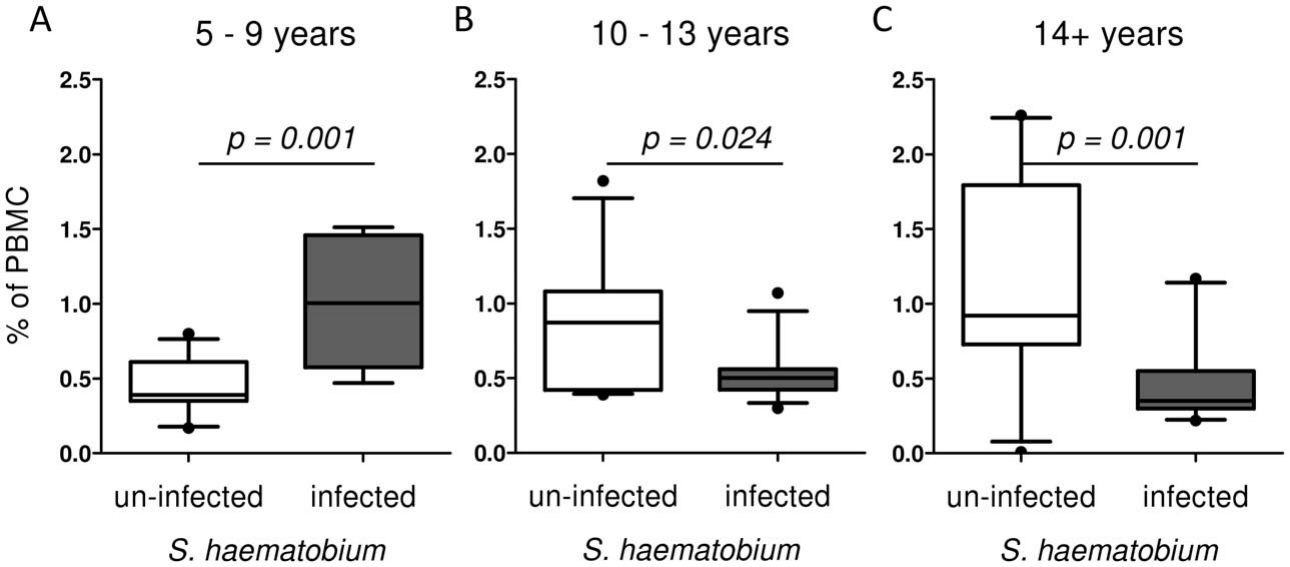
Proportions of mDCs in different age groups. The study population was divided into three age groups: 5–9 years (A), 10–13 years (B) and 14+ years (C). Proportions of mDCs were compared between un-infected (white box) and infected (gray box) individuals and expressed as % percentage of PBMC. Differences between un-infected and infected groups in the different age groups were analysed by univariate analysis after accounting for the effect of sex.

**Figure 6:**
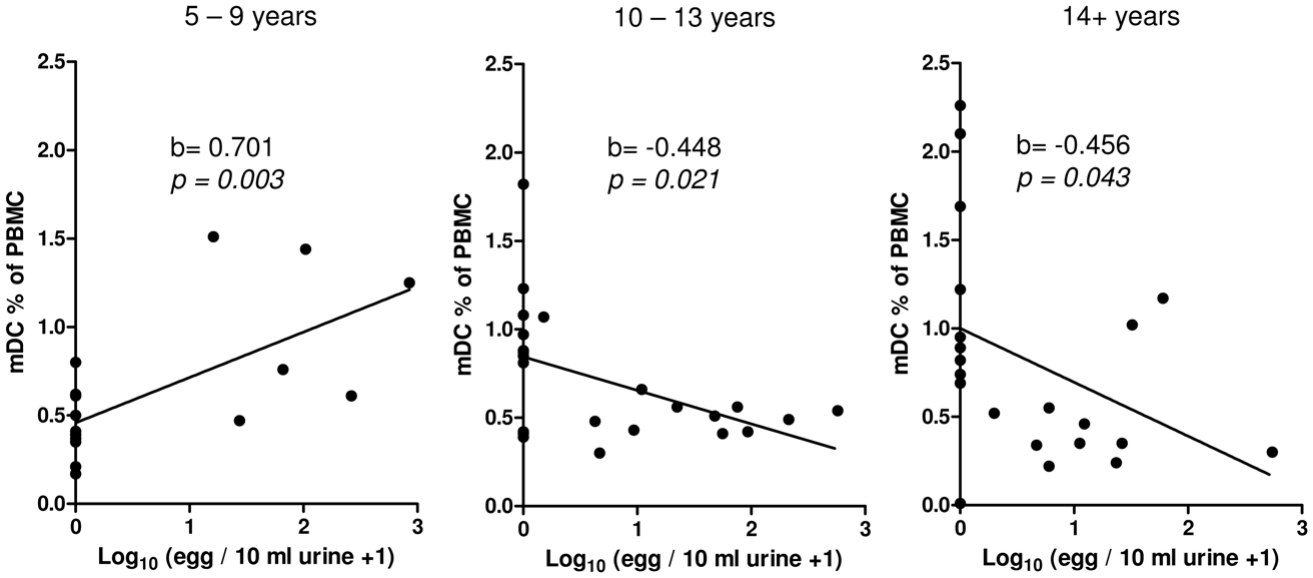
Correlation between infection intensity and mDCs. The study population was divided into three age groups: 5–9 years (left panel), 10–13 years (middle panel) and 14+ years (right panel). Proportions of mDCs expressed as % of PBMC are plotted against infection intensity (log_10_ (mean egg count+1) transformed). Correlation coefficient b and significant values of a correlation analysis after allowing for the effect of sex are indicated.

The age in the total population ranged from 5–45 years. Age group significantly affected the proportions of pDCs (Table 2), with a significant increase between 5–9 year olds (age group 1) and 14+ year olds (age group 3; p = 0.033). In contrast for mDCs the relationship with age varied depending on infection status (significant interaction between age group and infection status; Table 2). Based on these results post hoc analysis of mDC proportions by infection status was performed after partitioning the study population into the three different age groups (details in Table 1). In the youngest age group (Figure 5A) infected people had significantly higher mDC percentages than un-infected people. Results were comparable if expressed as percentage of CD14^neg^HLA-DR+ (Figure S1A). This pattern differed in the second age group, where infected individuals showed fewer mDCs than un-infected individuals (Figure 5B) a difference which was even more pronounced in the oldest age groups (Figure 5C). A comparable analysis could be made by correlating infection intensity (rather than infection status) to percentages of mDCs in the three different age groups. In the youngest age group both parameters were positively correlated (b = 0.707, p = 0.001), whereas in the second age group the correlation was instead negative (b = −0.441, p = 0.018). This negative correlation was more pronounced in the third group (b = −0.540, p = 0.007; Figure 6). A test for the homogeneity of the correlations coefficients showed a significant difference between age group one and two (z = 3.89, p = 0.0001) and between the first and the third age group (z = 4.17, p<0.0001).

**Figure 7:**
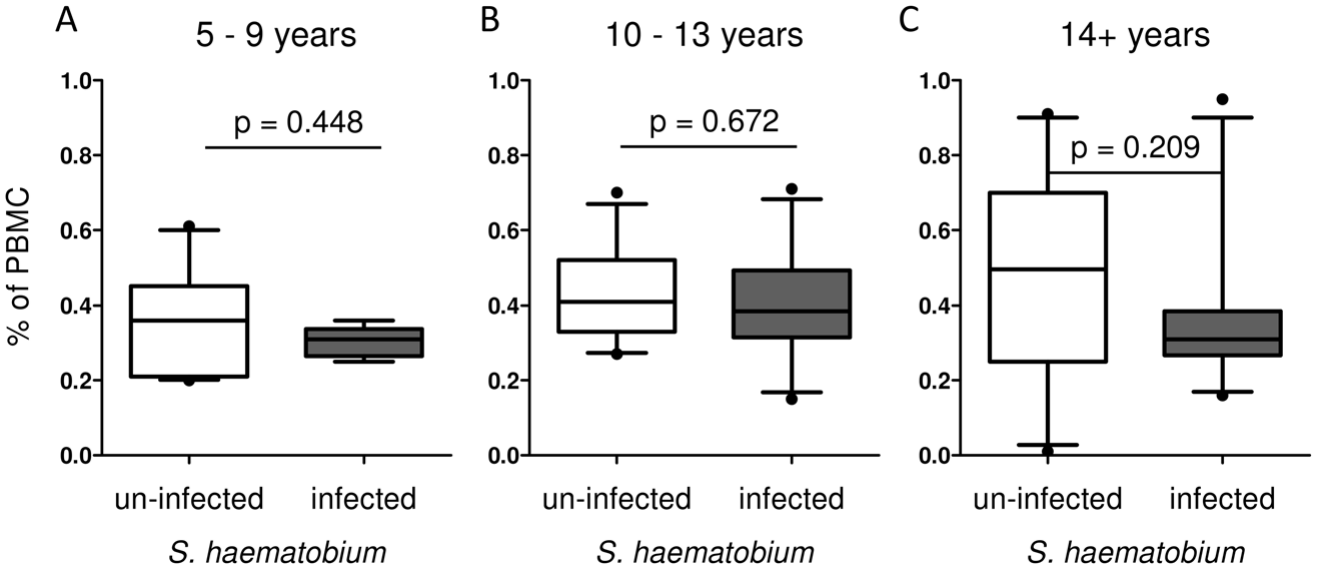
Proportions of pDCs in different age groups. The study population was divided into three age groups: 5–9 years (A), 10–13 years (B) and 14+ years (C). Proportions of mDCs were compared between un-infected (white box) and infected (gray box) individuals and expressed as percentage of PBMC (B). Differences between un-infected and infected groups in the different age groups were analysed by univariate analysis after accounting for the effect of sex.

In contrast, to the clear picture in the case of mDCs, there was no significant difference in the proportion of pDCs (expressed either relative to live PBMC or CD14^neg^HLA-DR+) between un-infected and infected people in any of the three age groups as shown in Figure 7, Figure S1B and Table 2.

### Expression of CD86 and HLA-DR on mDCs and pDCs

**Figure 8:**
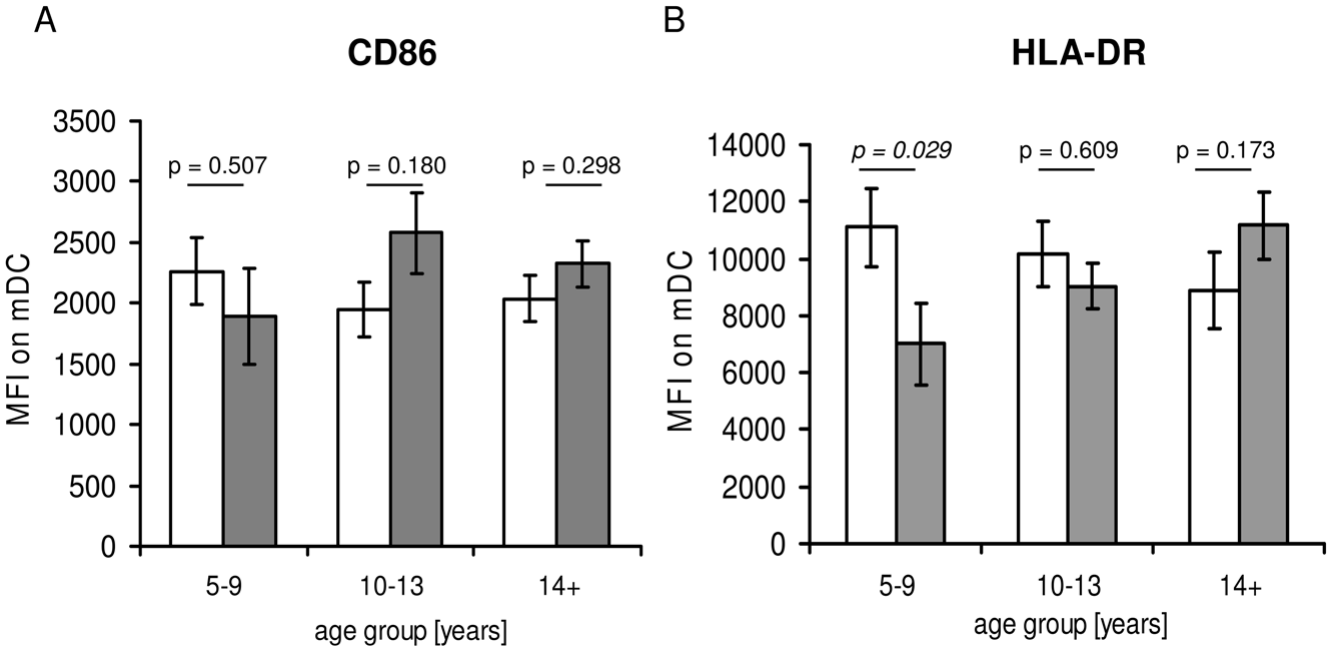
Expression levels of CD86 and HLA-DR on mDCs. PBMC were gated on mDCs as indicated in Figure 2 and analysed for the expression of CD86 (A) and HLA-DR (B). Data partitioned into three age groups as indicated and compared between un-infected (white columns) and infected individuals (gray columns).

Up-regulation of CD86 is hallmark of maturation and activation of DCs and their ability to provide co-stimulatory signals to T cells. HLA-DR expression can be used as an indicator both of DC activation status and their potential to activate antigen-specific T cells. Neither infection status nor the interaction between infection status and age group influenced expression of CD86 on mDC as determined by ANOVA (Table 2), which was confirmed by post hoc tests comparing CD86 expression between un-infected and infected people after partitioning into age group (Figure 8A). However HLA-DR expression was significantly influenced by an interaction of age group and infection status (Table 2). Post hoc test showed that HLA-DR expression differed between un-infected and infected individuals, in the youngest age group. In this age group HLA-DR was significantly lower on mDCs from infected people compared to un-infected (Figure 8B). In contrast no difference was observed in HLA-DR expression in people 10–13 years of age or in the oldest age group. Neither CD86 nor HLA-DR expression on pDCs were dependent on age group, infection status or the interaction of these variables (Table 2).

### Interaction between mDCs and WWH-IgE

**Figure 9:**
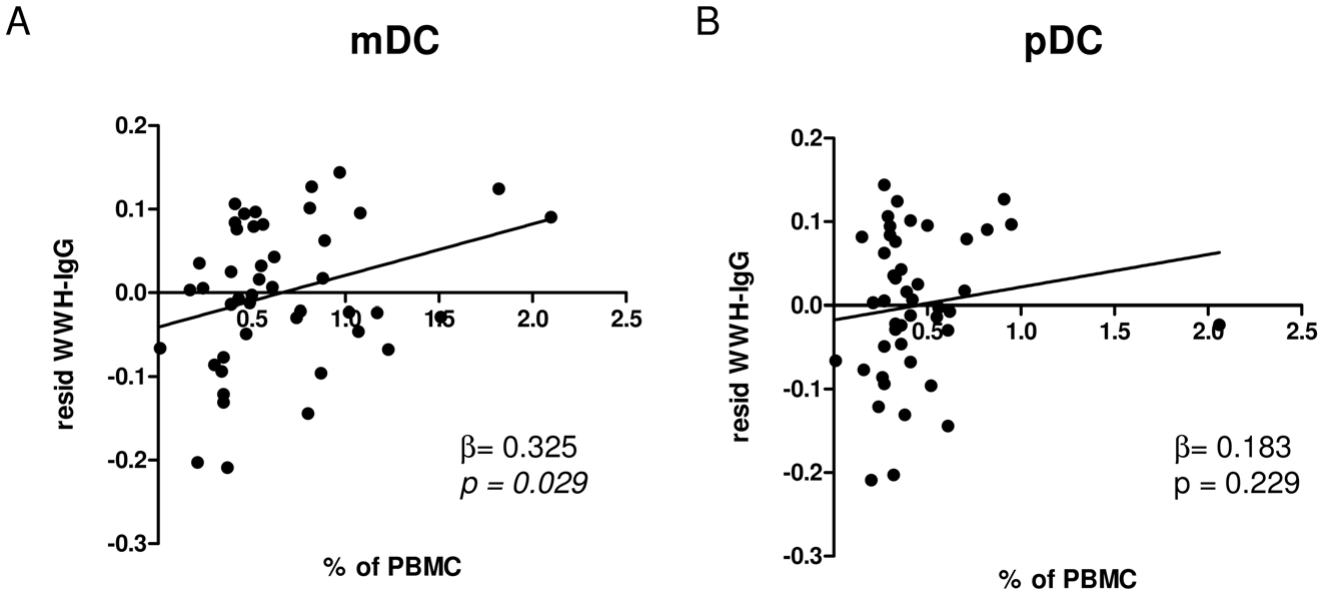
Correlation between WWH-IgG and proportions of mDCs and pDCs. Serum levels of WWH-specific IgG were measured by ELISA. The effects of sex, age and infection intensity was statistically allowed for and residuals of this analysis were plotted against proportions of mDCs (A) or pDCs (B) in percentage of PBMC. β-coefficient and p- values of a linear correlation are indicated.

Adult worm specific IgG can be used as a marker development of resistance against infection [59]–[61]. WWH-specific IgG increases with age and in our population especially between the first and second age group (Figure 2B) and to the same time changes in mDC proportions between un-infected and infected individuals occurred. Therefore the interaction between mDC and WWH-specific IgG was analysed. As indicated in Table S1, WWH-specific IgG is influenced by sex, age and infection intensity, but addition also by proportions of mDC. mDC are directly correlated to WWH-specific IgG after allowing for the effects of sex, age and infection (Figure 9A). Although showing the same tendency pDCs and WWH-specific IgG were not significantly correlated (Figure 9B). This correlation was most obvious in the oldest age group (Table S2) and overall more significant in un-infected (protected) individuals. Correlation between mDC proportions and WWH-specific IgE and IgM were not significant.

## Discussion

Urogenital schistosomiasis caused by *S. haematobium* shows a characteristic age-infection profile with increasing infections intensities during early childhood, peaking usually between the age of 9–15 years and than slowly declining towards adulthood [2]–[4]. This study investigated whether the age-related changes in schistosome infection pattern impacted on the proportions and phenotype of DCs.

We provide evidence that indeed there is an age-related pattern in the proportions of mDCs, if infected and un-infected people were compared. The proportion of mDCs were found to be present in significantly higher proportions in infected individuals compared to un-infected individuals in the youngest age group carrying heaviest infection levels. This subsequently changes in older age groups, were older infected individuals had lower relative numbers of mDCs compared to un-infected people. The gradual change between the three age groups became more apparent when the correlation between infection intensity (rather than infection status) and mDCs proportions were considered. In this case both parameters were positively correlated in the youngest age group and became a negative correlation with increasing age. This pattern is consistent with that predicted by quantitative studies for protective immune responses and has already been demonstrated for antibody responses associated with protection to re/infection in human hookworm [63] and schistosome infections [64]. Importantly levels of adult worm specific IgG are correlated with proportions of mDCs. This effect was more significant in the oldest age group, in which protective immunity becomes effective.

Our data confirm some of the major findings of a previous study of DC populations in human schistosomiasis conducted by Everts *et al* [32]. Proportions of mDCs were lower in an age group 14–45 years (mean 22.6 years) which is comparable to the population studied by Everts *et al* (17–39 years). Since cellular immune responses might depend on many different parameters such as transmission dynamics or genetic background of the investigated study population the consistency in DC proportions in schistosome-exposed human between our study and that by Everts *et al*, by itself is an important finding. A major difference between the two studies is that we did not observe differences in pDC percentages in regards to infection status, although we had high variation in pDC levels in the oldest age group. In deed a slight tendency of lower pDC proportions (expressed as percentage of PBMC) was observed in this age group. Differences in co-infections may also account for the differences between our observations and those of Everts *et al* [32] in Gabon. While the population in Zimbabwe had no co-infection with other helminths, Plasmodium or HIV, that in Gabon had co-infections with microfilaria observed in 48% of the schistosome infected individuals which could affect the pDCs proportions.

Both our study and that of Everts *et al* [32] clearly indicate the difference in mDC populations between infected and un-infected adults. However, since infections in moderate to high transmission areas occur at relatively young age, we were interested in the dynamics of the DCs over time, which was not possible in Evert's study. We wanted to determine when if at all the difference between infected and un-infected people was established. Therefore, we analysed DC cell populations in younger age groups. Our study demonstrates that a lower frequency of mDCs in infected people can be already observed in 10–13 year olds. This is accordance with changes in other immunological parameters as clearly shown for schistosome-specific IgG, IgE and IgM. The importance of DC in T_H_2 induction has been recently shown [23]. By altering cytokine responses DC could finally be involved in isotype switching and development of protective immunity. This is supported by our finding of the correlation between schisotosome-specific IgG and mDCs.

Changes with age have been also observed for serum cytokines in other study populations with moderate/high schistosome transmission [10], [11], [64]. For instance, high levels of parasite-specific or serum levels of IL-10 have been reported already at this age in different populations [11], [65] and might contribute to such changes. For example, DCs treated with the regulatory cytokine IL-10 inhibit T cell activation [66]–[69]. Earlier studies showed that T cell hypo-responsiveness can be already observed at early ages [70], [71]. The role of mDCs in induction of this impairment remains to be further investigated.

Other reasons for reduced relative numbers of mDCs in infected individuals in older age groups remain to be determined, e.g. enhanced apoptosis, reduced bone marrow output or increased migration to inflamed tissues or lymphoid organs were proposed by Everts *et al* [32]. Indeed enhanced migration in the context of T_H_2 priming DCs has been reported [25], [72]. This was induced by eosinophil-derived neurotoxin; a molecule elevated in serum of *S. haematobium* infected people [73]. Everts *et al* reported a more general impairment of DCs affecting both T_H_1 and T_H_2 cytokines as well as IL-10 in older individuals (17–39 years). The mechanisms involved are still unclear since despite this impairment of DC function, older individuals carrying schistosome infection can show enhanced immunopathology [74], [75] indicating an effective immune response.

In contrast, our data show that in the younger individuals (5–9 years) mDCs proportions were higher in peripheral blood of infected individuals in comparison to un-infected. Potential reasons for this include enhanced bone marrow output or reduced migration of DCs in the context of blood-born antigens [76]. Infection levels were highest in the youngest age group of our study population and young people might have more blood migrating schistosome stages affecting proportions of circulating mDCs. In contrast enhanced immune response including enhanced migration of mDCs might lead to increased killing of infiltrating schistosomula in older age groups.

Dendritic cell activation/maturation is characterised by up-regulation of cell-surface markers such as CD86 and MHC class II molecules. As previously reported by Everts *et al* [32], we observed no changes in expression of CD86 in infected people. However HLA-DR levels differed. In the youngest age group (5–9 years), where we found higher percentages of mDCs, expression levels of HLA-DR were lower in infected people. Reduced HLA-DR expression might be an indication of less matured and possibly fewer migrating cells, which subsequently could result in a less pronounced induction of T cell response in an early phase of exposure to schistosomes accompanied by a more regulatory environment due to Tregs and parasite-specific IL-10 [11], [12], [65]. Analysis of Tregs from another study cohort from the same population revealed a comparable pattern as recently published by our group [12]. In addition a direct comparison of mDCs and Tregs would need a larger sample size, since statistical power is lost by comparing the two parameters in the presence of other potentially confounding variables such as host age.

In contrast to the study by Everts *et al*, we did not observe a significant difference of HLA-DR levels in the older age groups. Again more significant changes of HLA-DR in these age groups might be due to cumulative effect of co-infections, not present in our study population.

Inhibition of up-regulation of activation markers such as CD86 has been suggested to be involved in induction of tolerogenic DCs [66]. However we did not see changes in the activation marker CD86. There is need to analyse other markers, such as B7-H1, B7-H2, lg-like transcripts 3 and 4 which might play a role in the function of tolerogenic DCs [77]–[81], particularly in younger age groups.

Functional studies, will be very informative and will clarify some of these areas, but were beyond the scope of this study.

In summary this study clearly shows there is an age-related pattern in the proportions mDCs in people exposed to *S. haematobium* infection as is typical for many already investigated humoral and cellular responses. Furthermore, in the case of mDCs, this pattern differs between schistosome infected and un-infected people and also follows the age profile with infection of protective responses, which is supported by the correlation between schistosome-specific IgG and proportions of mDCs.

The mechanism and consequences (such as alterations in the potential to induce different T cell phenotypes) behind the association between mDCs and infection remain to be determined. Understanding the nature and dynamics of immunological parameters in natural helminth infections and, in particular, changes of DCs involved in polarising the immune responses has an important impact on the development of vaccines against schistosomes.

## Supporting Information

Figure S1 **Proportions of mDCs and pDCs expressed as percentage of CD14neg-HLA-DR+ cells.** The study population was divided into three age groups: 5–9 years (A), 10–13 years (B) and 14+ years (C). Proportions of mDCs (A) or pDC (B) were compared between un-infected (white box) and infected (gray box) individuals and expressed as % percentage of CD14neg-HLA-DR+. Differences between un-infected and infected groups in the different age groups were analysed by univariate analysis after accounting for the effect of sex.
(TIF)

Table S1 **Interaction between WWH-IgE and mDC or pDC.**
(TIF)

Table S2 **Correlation between WWH-IgE and mDC or pDC.**
(TIF)
