# Supplementary figures and images for: Age-Related Patterns in Human Myeloid Dendritic Cell Populations in People Exposed to Schistosoma haematobium Infection
Source: PLoS Negl Trop Dis. 2012 Sep 27;6(9):e1824. doi: 10.1371/journal.pntd.0001824 (PMC3459871; doi:10.1371/journal.pntd.0001824)

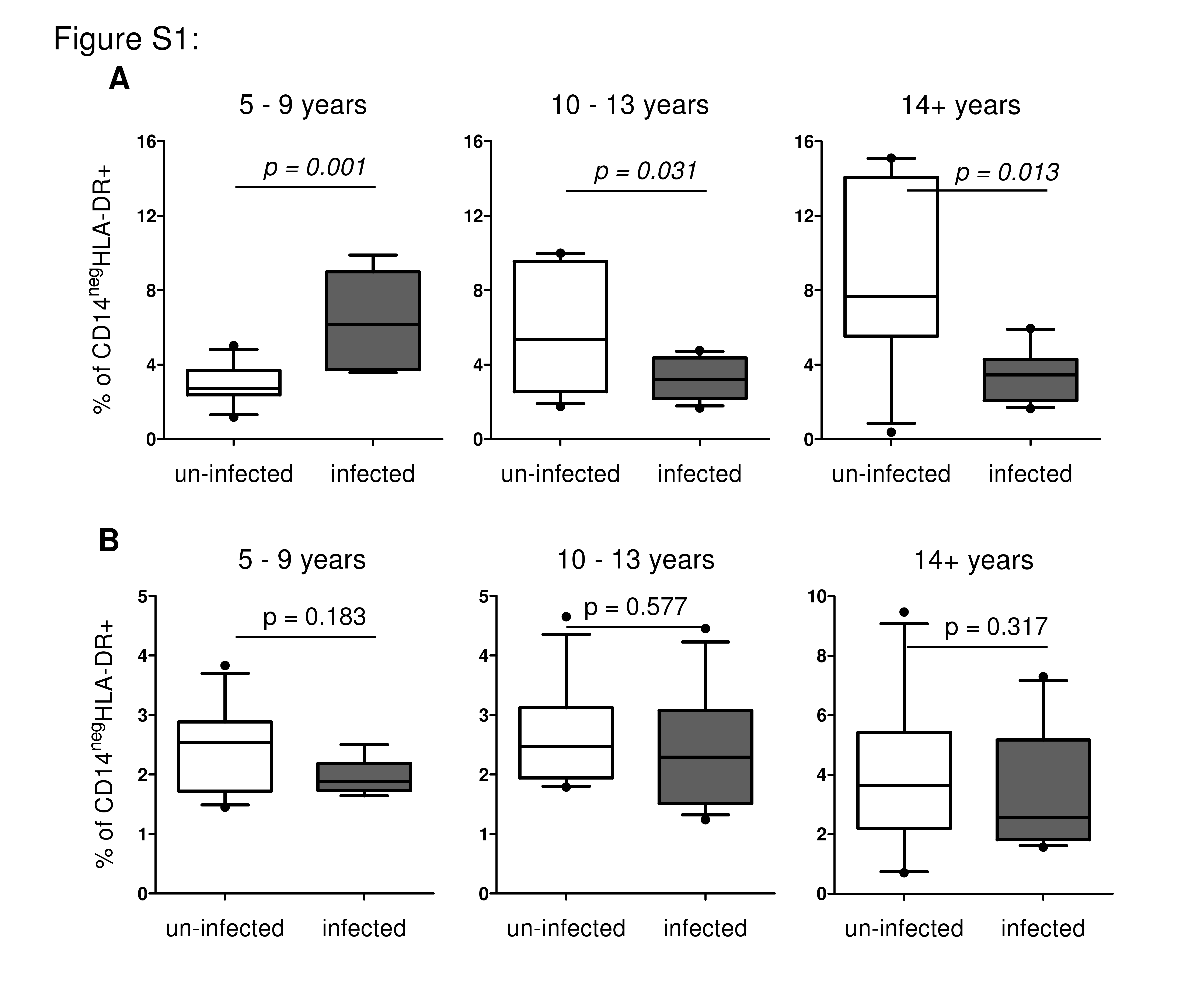

Supplement: Figure S1 — Proportions of mDCs and pDCs expressed as percentage of CD14neg-HLA-DR+ cells. The study population was divided into three age groups: 5–9 years (A), 10–13 years (B) and 14+ years (C). Proportions of mDCs (A) or pDC (B) were compared between un-infected (white box) and infected (gray box) individuals and expressed as % percentage of CD14neg-HLA-DR+. Differences between un-infected and infected groups in the different age groups were analysed by univariate analysis after accounting for the effect of sex. (TIF) [file pntd.0001824.s001.tif]

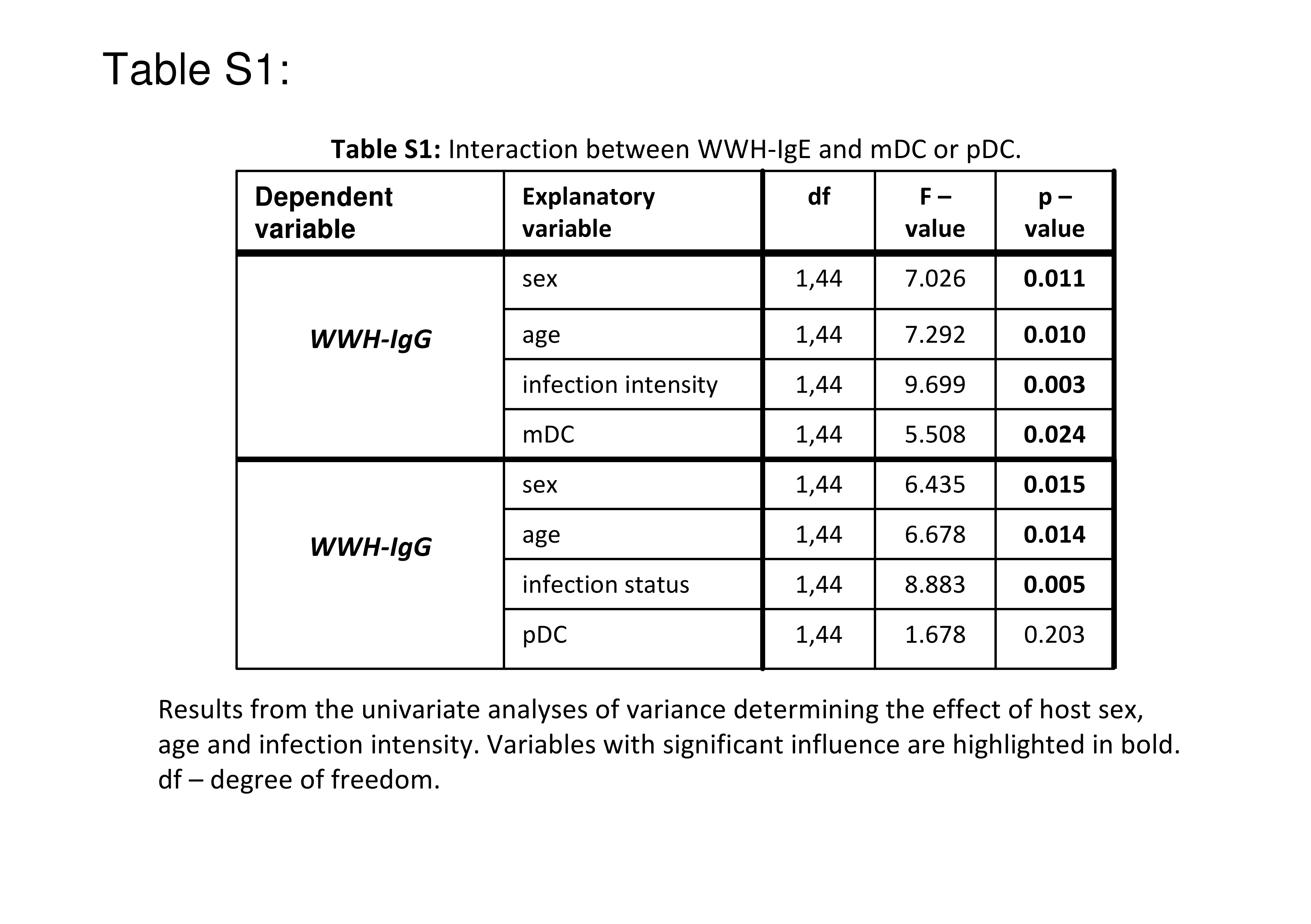

Supplement: Table S1 — Interaction between WWH-IgE and mDC or pDC. (TIF) [file pntd.0001824.s002.tif]

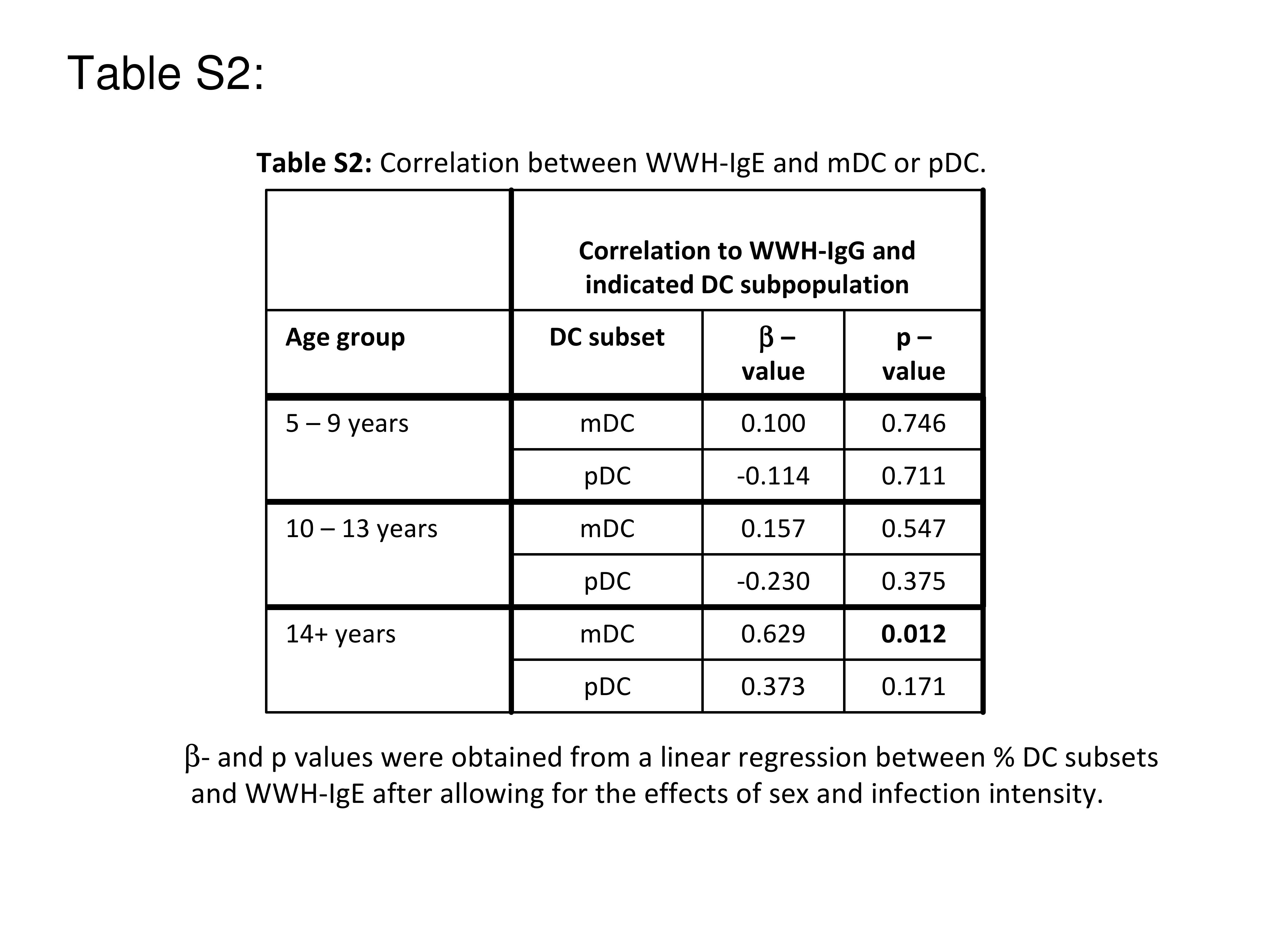

Supplement: Table S2 — Correlation between WWH-IgE and mDC or pDC. (TIF) [file pntd.0001824.s003.tif]
